# Robotic “Zero Contact” surgery for occupational protection against infectious disease

**DOI:** 10.3389/fpubh.2022.977927

**Published:** 2022-10-17

**Authors:** Yiling Shen, Rongrong Ge, Xinye Qian

**Affiliations:** ^1^Department of Surgical Operating Room, Xinhua Hospital Affiliated to Shanghai Jiao Tong University School of Medicine, Shanghai, China; ^2^Department of Plastic Surgery, Wuxi Dashangmei Plastic Surgery Hospital, Wuxi, China; ^3^Center of Hepatobiliary Pancreatic Disease, Beijing Tsinghua Changgung Hospital, School of Clinical Medicine, Tsinghua University, Beijing, China; ^4^School of Clinical Medicine, Tsinghua University, Beijing, China

**Keywords:** infectious disease, surgical team, Zero Contact, robotic surgery, COVID-19

## Introduction

The COVID-19 pandemic has affected almost every aspect of the medical system, including a group of medical workers who seem to have the least relationship with COVID-19 — surgical teams (1).

Nowadays, surgery is an essential treatment for diseases like tumor, trauma, etc. Because of this, medical centers all over the world are trying their best to provide surgical interventions for patients in need during the COVID-19 pandemic (2). However, this would make surgical team members vulnerable to COVID-19 infection as surgical patients might be COVID-19 patients, suspected cases or close contacts. In fact, surgical team members are constantly exposed to infectious diseases (3), including HIV, hepatitis B, tuberculosis, etc., when they are performing surgeries for patients with infectious diseases, which might seriously affect the occupational health and safety of surgical team members.

Infection of surgical team members during surgery could not only bring disaster to the medical workers both physically and mentally, but also bring disaster to the hospital (like nosocomial infection spread by doctors, which has been reported worldwide during the COVID-19 pandemic, including China).

## The concept of “Zero Contact”

Three pillars of infectious diseases are source of infection, route of infection, and susceptible people. Source of infection refers to people and animals who have pathogens growing and reproducing in their bodies and have the ability to excrete these pathogens, including patients, pathogen carriers and infected animals. Route of infection refers to the process that pathogens are discharged from the source of infection and invade susceptible people through a certain mode of transmission. For example, airborne transmission is the main mode of transmission of respiratory infectious diseases, including droplet transmission, etc. Susceptible people refers to the people who lack immunity to a certain infection and are vulnerable to the disease.

If the route of infection could be completely cut off, the infection disease would be controlled immediately. Accordingly, the concept of “Zero Contact” has been developed, which refers to the complete separation of uninfected personnel from infectious sources in space so as to prevent the spread of infectious diseases. Although it is an ideal concept, many epidemic prevention strategies are originated from it, such as quarantine (4), facial mask, etc.

## Current protection for surgical team

**Figure 1:**
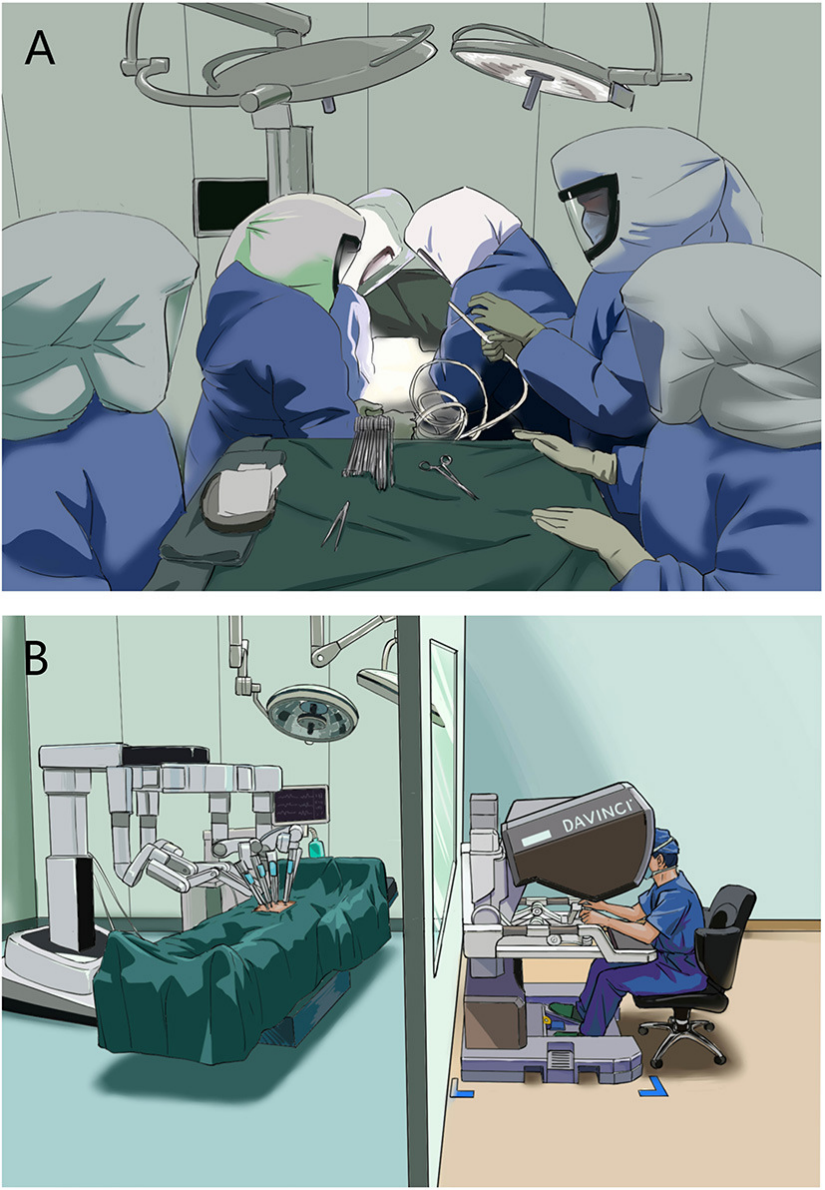
Surgery for patients with infectious disease. **(A)** Traditional protective surgery for infectious disease. **(B)** Robotic “Zero Contact” surgery for infectious disease.

In guidelines and regulations for infectious operation [surgical patients with infectious diseases, like COVID-19 (5)], personal protective equipment (PPE), such as isolation gown, facial masks, goggles, etc, is one of the most important protective measures (Figure 1A).

The rationale behind these measures is the concept of “Zero Contact” since almost every existing measure is trying to isolate infection sources from medical workers so that the infection would not happen. However, these methods still have disadvantages in practice. First of all, protective equipment could pose a great challenge to the physical fitness of the surgical team. Antonio Scarano et al. reported that protective facial mask could induce a reduction in circulating oxygen concentrations, an increase of heart rate and a sensation of shortness of breath (6). Thiagarajan et al. also pointed out that protective equipment would make the surgical team feel uncomfortable (7). Eye protection measures could even lead to vision problems (8). Under these conditions, technical deformation and prolonged operation time could happen, resulting in increasing possibility of accidental injury during operation, such as suture needle injuries, electrosurgical unit injuries, etc. On one hand, these injuries could lead to the infection of medical workers both directly (the exchange of blood between medical workers and patients) and indirectly (the damage of protective equipment). On the other hand, these injuries could cause surgical complications (such as bleeding, tissue damage, etc.) in surgical patients, affecting their prognosis.

Moreover, the COVID-19 has exposed other drawbacks of these methods. First, some variants of the COVID-19 (like Delta and Omicron variants) are extremely contagious; they might infect medical workers wearing PPE. As the diameter of the virus is around 0.07–0.09 μM, FFP3 masks could filter over 99% for 8 h; FFP2 or N95-equivalent respirators could filter over 92–98% for 8 h; standard surgical masks could only filter 63% for 30 min. Therefore, no mask could provide definite protection. In addition, if the mask could not fully fit the face, it would also increase the probability of infection (9). Second, COVID-19 virus could persist on inanimate surfaces for up to 9 days (10). Surgical team members might be infection if they made mistakes when taking off protective equipment. Third, these protective methods need large amount of medical resources, which have been rapidly overwhelmed in the short history of COVID-19, including PPE. As a result, many institutions have to downgrade the protection level for surgical teams, such as using only surgical masks instead of FFP3 masks.

## “Zero Contact” operation based on robotic surgical system

Robots, one of the novel technologies raised in this pandemic, play an important role in distributing medical services, including monitoring patients, sanitizing hospitals, making deliveries, and helping frontline medical workers reduce their exposure to the virus (11). Using robots as the communication medium between doctors and patients could completely avoid the direct contact between medical staff (susceptible person) and patients (potential infection source), realizing the concept of “Zero Contact.” Besides, robots could be thoroughly disinfected repeatedly.

In fact, the surgical association already has a system that could realize “zero contact” operation, namely surgical robot system. The system was originally designed for remote surgery. In 1996, Dr. Jon Bowersox completed the first remote robotic surgery (12). Yet, this concept of tele-surgery of the robotic system has almost been abandoned these years and replaced by the concept of “minimal invasive surgery” (13). Although long-distance robotic tele-surgery would still face problems such as data transmission and surgical safety, the current robotic surgery system could fully realize short-distance remote control. Also, surgical safety could be guaranteed because the whole surgical team is nearby. Based on these features, the surgical team could perform surgery in a room isolated from the operating room to achieve “Zero Contact” surgical treatment (Figure 1B), thus protecting the surgical team from infection. This robotic “Zero Contact” surgical system might solve the shortcomings of traditional surgical protection methods for patients with infectious diseases, especially during pandemic like COVID-19.

1. The robotic “Zero Contact” surgery system could provide complete protection for surgical team members as it could block the contact between the surgical team and the source of infection. In addition, surgical teams do not need to worry about infection happened when they take off the protective equipment as PPE is no longer required.
2. This robotic surgery system could greatly reduce the consumption of protective equipment. Thus, the pressure of medical supplies shortage caused by infectious diseases (such as COVID-19) could be relieved.
3. This robotic surgery system could also ensure the quality of surgery. As surgical teams are free of protective equipment, they would be more comfortable than performing the surgery in heavy protective armors. Vision would not be damaged since goggles are not needed. Furthermore, the development of robotic surgery has ensured that the system could complete almost all kinds of abdominal and thoracic operations with standard quality, including vascular anastomosis (13).

However, robotic “Zero Contact” surgery system still has a long way to go. Robotic anesthesia system (14) needs further improvement. Similarly, assistant robots should be developed as current robotic surgery still needs human assistants to prepare surgical instruments and replace mechanical arms. Additionally, robotic surgery is mainly used for thoracic and abdominal surgery; its application in other area like trauma, neurosurgery, etc, needs further validation and discussion.

## Conclusions

The COVID-19 pandemic has made the entire medical community realizing that there are still many problems in the existing system. Only by taking precautions can we be able to deal with the disaster when it comes again. Although there are still many problems to be solved, the robotic “Zero Contact” surgery system could protect the surgical team from the threat of infectious diseases and provide high-quality surgical treatment for patients.

## Author contributions

YS and XQ conceived of the project. YS and RG wrote the paper. XQ provided expert guidance and suggestions. All authors contributed to the article and approved the submitted version.

## Funding

This work was supported by the Talent Researchers of Tsinghua University (No. 10001020507).

## Conflict of interest

The authors declare that the research was conducted in the absence of any commercial or financial relationships that could be construed as a potential conflict of interest.

## Publisher's note

All claims expressed in this article are solely those of the authors and do not necessarily represent those of their affiliated organizations, or those of the publisher, the editors and the reviewers. Any product that may be evaluated in this article, or claim that may be made by its manufacturer, is not guaranteed or endorsed by the publisher.
